# Increased aluminum exposure induces widespread changes in silicon, carbon, and nitrogen metabolism in *Entomoneis vertebralis*

**DOI:** 10.1186/s12864-025-12106-7

**Published:** 2025-10-16

**Authors:** Ramya Ragunathan, Hugh M. Purdy, Susanna Seppala, Hosu Gwak, Sara Calhoun, Benjamin S. Twining, Igor V. Grigoriev, Bradley F. Chmelka, Mark A. Brzezinski, Michelle A. O’Malley

**Affiliations:** 1 Department of Chemical Engineering, University of California, Santa Barbara, CA 93106 USA; 2 Department of Ecology, Evolution, and Marine Biology, University of California, Santa Barbara, CA 93106 USA; 3 Bigelow Laboratory for Ocean Sciences, 60 Bigelow Drive, East Boothbay, Maine, 04544 USA; 4 U.S. Department of Energy Joint Genome Institute, Lawrence Berkeley National Laboratory, 1 Cyclotron Rd, Berkeley, CA 94720 USA; 5 Department of Plant and Microbial Biology, University of California Berkeley, 110 Koshland Hall, Berkeley, CA 94720 USA; 6 Department of Bioengineering, University of California, Santa Barbara, CA 93106 USA

**Keywords:** Algae, Diatom, Biomineralization, Aluminum, Metabolism, Structure-based similarity, Transcriptomics

## Abstract

**Background:**

Diatoms are a class of algae that play an essential role in global ecology and produce valuable chemicals. They are known for forming intricate nanostructured silica cell walls (frustules). The introduction of non-siliceous elements like aluminum into diatoms induces properties such as a lower dissolution rate of the frustule, increasing the specific surface area of the frustule and enhancing metabolism. Previous studies have focused primarily on characterizing physiological impacts, leaving the genetic response(s) to non-siliceous elements largely unexplored.

**Results:**

This study investigates the transcriptional response of the pennate diatom, *Entomoneis vertebralis* to dissolved aluminum. Our findings reveal that in the presence of added 10 µM aluminum, biogenic silica content of the cell wall increases approximately twofold along with significant changes to core metabolism. An increase in transcription of genes encoding nitrate transporters has been observed despite the apparent downregulation of nitrogen assimilation pathways. Additionally, increased transcription of genes involved in carbon fixation were noted. Amino acid and protein motif analyses identified proteins that were differentially regulated shared amino acid compositions and motifs characteristic to silicification-associated genes. A unique structure-based analysis pipeline revealed that some of these proteins have a conserved structural core while being diverse in sequence, which could be features associated with biosilicification.

**Conclusion:**

Differential expression transcriptomics has provided insight into diatom metabolism when exposed to aluminum, highlighting potential targets for metabolic engineering. Furthermore, we identified potential silicification-associated genes using tools based on structure and amino acid composition, advancing our understanding of diatom silicification.

**Supplementary Information:**

The online version contains supplementary material available at 10.1186/s12864-025-12106-7.

## Introduction

Diatoms are a large group of unicellular photosynthetic eukaryotes that contribute 20% of Earth’s global primary production of organic matter, comparable to the total amount fixed carbon by all the terrestrial forests combined [1]. Diatom metabolism plays an important role in the cycling of many macronutrient elements, such as carbon, nitrogen, silicon, oxygen and phosphorus [2]. In addition, diatoms are known to produce valuable products such as lipids [3] and they synthesize complex, genetically encoded, hierarchical patterns of amorphous silica in their cell walls under environmentally benign conditions [4].

The silicified diatom cell wall, known as the “frustule”, exhibits a broad variety of morphologies [5], yet the molecular mechanisms underlying the formation of bio-silica structures remain elusive. This growing area of research is partly motivated by the potential to develop biological or bio-inspired methods that produce silica structures using sustainable conditions or inexpensive production platforms [6, 7]. Mechanistic and biosynthetic insights gained from studying the diatom’s unique metabolism could guide the engineering of diatoms to synthesize valuable products or customized silica-based structures for diverse applications, including drug delivery, membrane separations, and waste management [8, 9]. These efforts would be aided by an improved understanding of the response of diatom core metabolism to manipulations that affect silica deposition and structure formation to enable processes that achieve the desired response while maintaining cell viability and growth.

Attempts have been made to modify the diatom’s frustule by incorporating different non-silicon elements, such as titanium or germanium, to induce modifications with desirable properties [10, 11]. Among these, adding aluminum to frustules has resulted in favourable material properties, such as resistance to hydrolysis and higher specific area [12, 13], in addition to providing beneficial metabolic effects, such as enhancing the carbon fixation process [14]. There is some evidence that this enhancement is due to the ability of aluminum to form complexes that facilitate the conversion of specific ions, such as iron, into bioactive Fe^2+^ states [15] that are readily consumed by diatoms. Additionally, it has been demonstrated that aluminum increases the activity of alkaline phosphatases, enabling enhanced use of organic phosphorus, including dead biomass, which is typically not consumed by diatoms [16] and that could impact bio-silicification. Overall, these findings imply that aluminum influences an array of metabolic and biosynthetic processes in diatoms, including nutrient uptake and utilization of macronutrients, and could have significant implications for understanding the bio-silicification process and the intricate interplay between elements in marine ecosystems.

Aluminum incorporation experiments have been conducted in various diatom species [13, 17–20], revealing the ability of aluminum to alter silica content or morphology. Aluminum-induced changes might differ based on whether they are centric or pennate diatoms; however, it is subject to further verification. The pennate diatoms *Navicula salinarum* [20] and *Craspedostauros* sp. [13] showed increased biosilicification in the presence of aluminum. Similar studies on the centric diatom, such as *Thalassiosira weissflogii* showed no morphological change, but phosphate metabolism was affected [16]. The centric diatom *Thalassiosira pseudonana* also exhibited no morphological change in response to aluminum, but an increase in the specific area of the silicified cell wall was reported [19]. Further insights into the possible links between silicon metabolism and aluminum come from studies of higher plants that produce silica nanospheres to alleviate aluminum toxicity [21, 22]. There has been some evidence that adding aluminum causes modifications in frustule morphology, as discussed, which varies among different classes of diatoms; however, further studies in different types of diatoms are required to understand the species-specific impacts.

Understanding how metals such as aluminum are incorporated into diatom frustules and their impacts on silica content or morphology is currently limited by incomplete understanding of silica biosynthesis. Extensive research investigating the organic components associated with bio-silicification has led to the discovery of many protein families involved in this process, such as silaffins [23], silacidin [24], cingulin [25], and silicanin [26], through the analysis of transcriptomic changes during building the silica cell wall or by conducting proteomics on extracted frustules. However, none of these studies captured the transcriptomic changes that a diatom undergoes as its frustule morphology is altered in response to an environmental cue [5, 27]. It should be noted that several of these protein families, particularly the silaffins and silacidins, although characterized in the well-studied diatom *T. pseudonana*, are not conserved even among diatoms from the same genus [28]. As previously discussed, aluminum has induced frustule-associated morphological change in certain diatoms, which may affect the expression of silicification-related genes. Therefore, merging an understanding of transcriptomic shifts and corresponding biosilicification changes in diatoms exposed to aluminum could implicate genes involved in the morphological changes.

How the incorporation of non-siliceous elements, such as aluminum, affects the processes used by diatoms to construct their intricate silica-based cell wall remains unknown. Still, it likely involves many genes and their constituent protein products [5] from various metabolic pathways involving nutrient uptake and silica polymerization. An understanding of these impacts would help identify potential targets to metabolically engineer frustule morphology and, at the same time, elucidate biosilicification mechanisms and processes. An increase in bio-silicification in the presence of aluminum has been observed, particularly in pennate diatoms as mentioned previously. Here we examine the effect of aluminum on *Entomoneis vertebralis,* a newly sequenced pennate diatom, was used in this study to investigate the impact on its bio-silicification and overall metabolism.

In the present study, the impact of aluminum on the bio-silicification process(es), as well as its impact on core metabolism, are examined. Exposing a newly sequenced pennate diatom *Entomoneis vertebralis* to aluminum increased the amount of silica per cell. The transcriptomic response revealed altered carbon and nitrogen metabolism in the presence of aluminum and its impact on the expression of genes involved in silicification, paving the way to identify metabolic targets to induce desirable properties and valuable products.

## Results

### Entomoneis vertebralis has a unique cell wall morphology, and the addition of aluminum leads to an increase in silicification

**Fig. 1:**
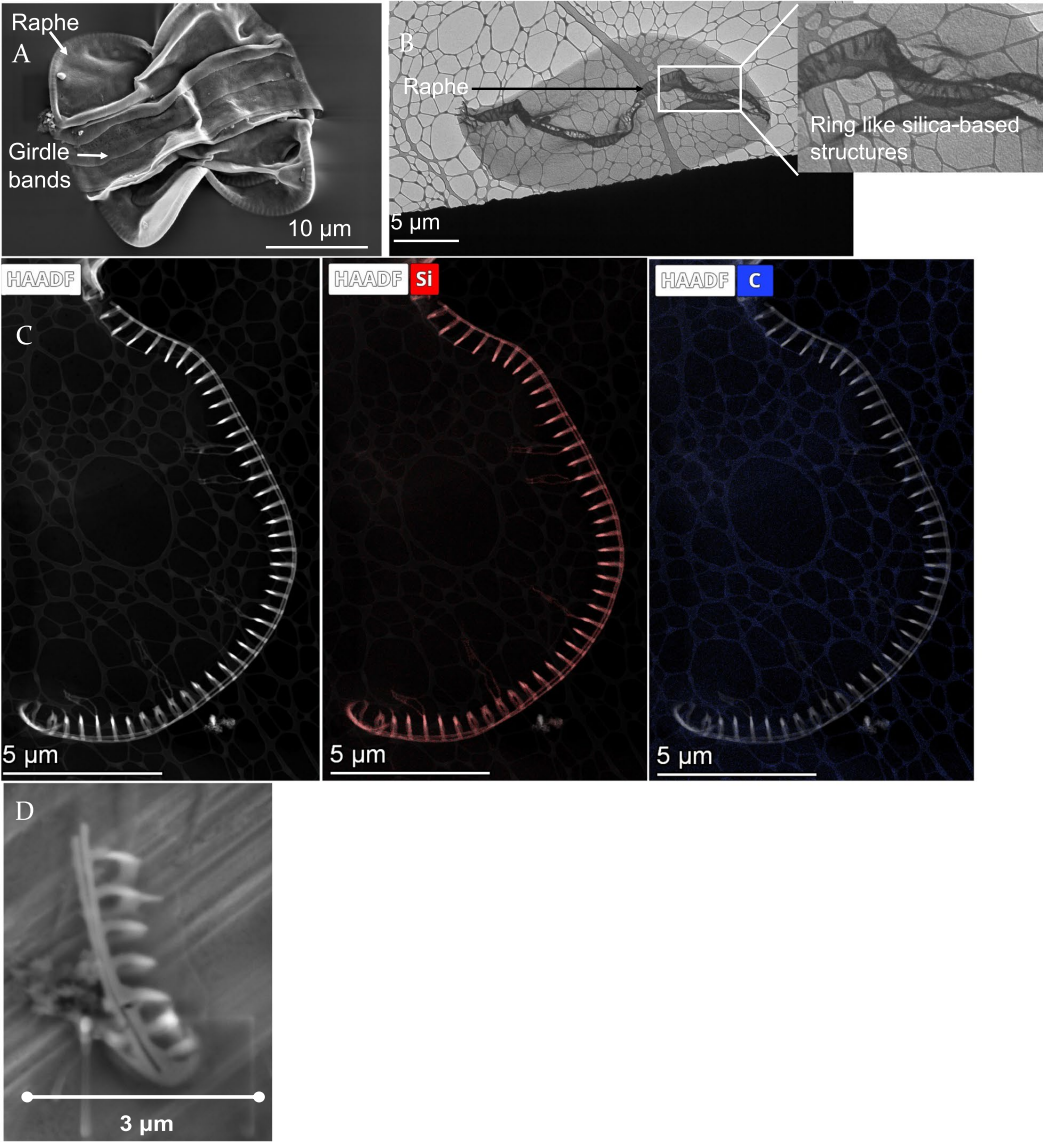
**A.** SEM image of Entomoneis vertebralis depicting the overall frustule morphology, with ring-like structures in the ralphe (outermost border as depicted in the picture). **B.** Top view of H_2_O_2_-cleaned frustule of Entomoneis vertebralis as seen in TEM, showing that the raphe majorly consists of ring-like structures. **C.** TEM-EDX on *Entomoneis vertebralis*, the composition of carbon and silica, shows that most of the silica was concentrated on the protruding circles and the rest of the cell wall is likely composed of carbon rather than silica. **D.** SEM image of a broken raphe showing the circular silica rings protruding from the raphe

The morphology of *Entomoneis vertebralis* frustule was examined using scanning electron microscopy (SEM) and transmission electron microscopy (TEM), as shown in Fig. 1. SEM characterization (Fig. 1A) revealed key structural features, including a raphe, valves, and girdle bands. TEM characterization (Fig. 1B) provided a top view of a broken valve, highlighting the raphe’s ring-like structures. The ring-like silica structure can be further seen when the raphe was disengaged from the rest of the frustule by sonication, as shown in Fig. 1D. These ring-like formations appeared to be the primary silicified regions of the valve, as confirmed by EDX-TEM analysis (Fig. 1C). In contrast, the rest of the valve was likely composed predominantly of organic matter, differing from the silica-rich composition typically observed in other silicified diatoms.

To understand this silicified frustule morphology, *E. vertebralis* was exposed to varying concentrations of aluminum [29]. Biogenic silica content was measured to understand the impact of increased aluminum on diatom silicification [30].

**Fig. 2:**
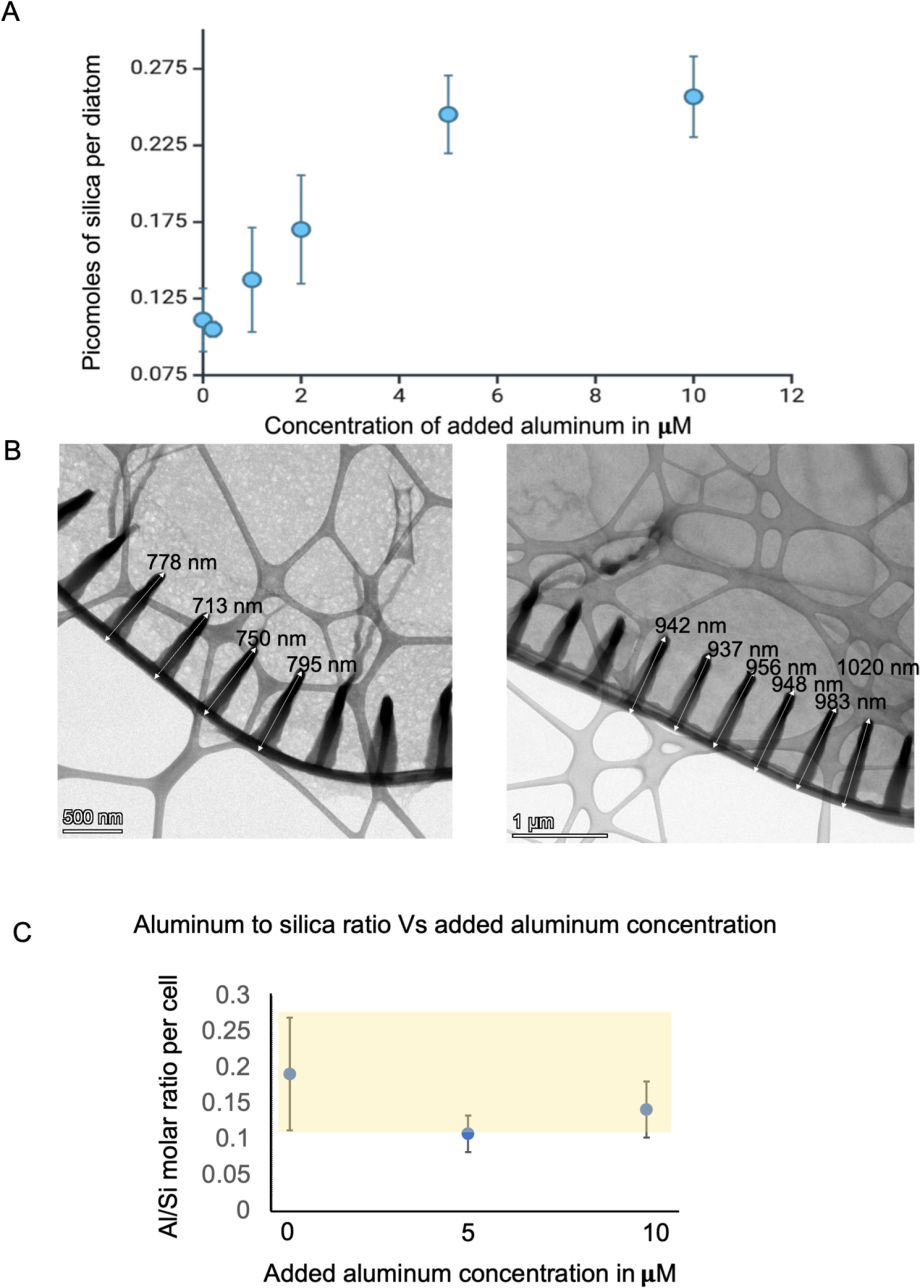
**A.** Effect of aluminum on biosilicification of Entomoneis vertebralis. As the amount of aluminum added was increased, the amount of silica per cell increased, however it saturated after the 5 µM aluminum scenario as indicated in the shaded box. **B. **TEM images of no aluminum (right) versus 10 µM aluminum exposed diatoms (left). Enlarged circles protruding from the raphe relative to the condition without aluminum were observed. **C.** Al/Si molar ratios for different concentrations of aluminum added to the media shows that there was no significant change in Al/Si ratio as depicted in the yellow box. Here aluminum refers to amount of aluminum present inside the cell, measured using ICP-MS

As shown in Fig. 2a, the silica content of *E. vertebralis* cells increased almost twofold in cultures that were exposed to 10 μM aluminum compared to the control, which was not supplemented with aluminum. Concentrations above added 5 μM aluminum did not lead to additional increase in silicification. ICP-MS revealed some amount of aluminum is present in diatoms from control cultures (Fig. 2c), suggesting potential aluminum background levels from the salts, that are part of the artificial seawater formula. Hereafter, any mention of aluminum concentration refers to the amount of aluminum added to the culture medium, if not specified. Transmission electron microscopy (TEM) revealed that the increase in silica content was accompanied by changes in frustule morphology, whereby with 10 μM aluminum, the frustule developed larger (From ~ 750 nm to ~ 950 nm) ring-like silicified structures within the raphe (Fig. 2b), which is the dominant silicified feature of *E. vertebralis*. Despite the increase in biogenic silica content with added aluminum, there was no noticeable change in the aluminum/silicon molar ratio across different concentrations of added aluminum to cultures, as shown in Fig. 2c.

### RNA-seq analysis of*E. vertebralis*exposed to high aluminum levels reveals significant alterations in primary metabolism

**Fig. 3:**
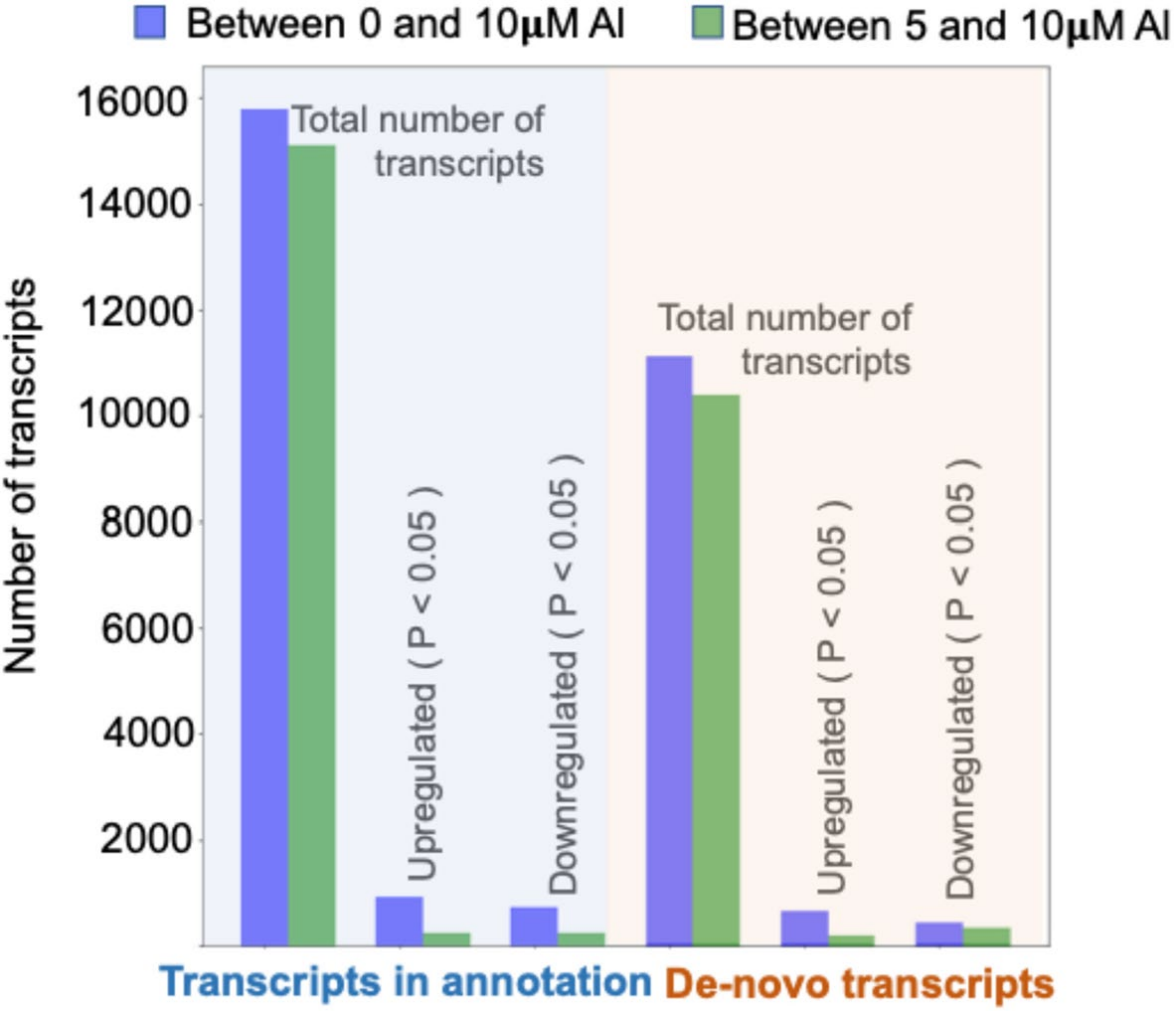
RNA-seq data characterizing the statistics of genes that were differentially expressed in Entomoneis vertebralis between 0 and 10uM aluminum (blue) and 5 and 10 uM aluminum (green), in both the classes, i.e. transcripts that are aligned to the genome (denoted in blue) and de-novo transcripts discovered using StringTie (denoted in red). “Total number of transcripts” indicates the total number of transcripts that were expressed with cpm > 0.5 in that case. Significantly upregulated and downregulated genes were chosen based on the adjusted p-value < 0.05. The adjusted p-value was calculated using edgeR (Benjamini-Hochberg (BH) method). There were more genes differentially regulated between 0 and 10 μM relative to the 5 and 10 μM scenario

RNA-seq was conducted utilizing the *Entomoneis vertebralis* reference annotation from JGI Phycocosm [31] to analyze the transcriptional response of this diatom towards supplemental aluminum. The dataset utilizing the transcript reads from samples exposed to different concentrations of added aluminum (5 μM and 10 μM) and a control (0 μM) indicated transcription of 44,665 genes based on the StringTie-generated [32] gene count matrix. However, not all StringTie-generated transcripts were present in the reference genome annotated through JGI Phycocosm [31], suggesting the presence of novel transcript structures not identified by the reference transcriptome. The results from the StringTie-generated gene count matrix, as shown in Fig. 3, indicate that more genes are differentially expressed between 0 μM and 10 μM aluminum cultures, compared to 5 and 10 μM aluminum cultures, in addition to new de novo transcripts observed in this study. The differentially expressed genes between 0 μM and 10 μM aluminum consisted of 1650 sequences, of which 818 had some predicted annotation (GO, InterPro, HMMER, Pfam, or KEGG, Supplementary Information). Analysis of the annotations indicated that aluminum affected gene expression across a diverse set of metabolic processes.

Biochemical assays indicated that there was a significant increase in silicification between diatom cultures without supplemental aluminum and those exposed to 5 μM or 10 μM of aluminum. However, there was no significant difference in silicification between cultures that were treated with 5 μM aluminum and cultures that were treated with 10 μM aluminum (Fig. 2). To focus on identifying responses associated with changes in silicification, we used these differences in silica content to isolate transcriptomic changes related to shifts in silicification. To identify genes involved in silicification, transcripts that were differentially expressed in 0 μM and 10 μM aluminum, but were not differentially expressed between the 5 μM and 10 μM aluminum treatments, were analyzed. Enzymes participating in polyamine synthesis were upregulated in the presence of added aluminum. Among these was Protein ID 999264, which encodes a putative homospermidine synthase. Protein ID 1041712 encodes what appears to be a fusion protein that contains a spermidine synthase domain as well as a SAM decarboxylase domain [33]. It has been suggested that these fusion proteins are unique to diatoms and that they are involved in synthesizing long-chain polyamines [33]. Furthermore, several serine proteases (subtilase family) were downregulated in higher aluminum conditions (Protein IDs 1050279,1087708, and 1087724). Various ubiquitin ligases also appeared to be significantly downregulated (Protein IDs 1003455, 1070178, 626300, 814710, 960722, 970818, and 1073132), while some were upregulated (Protein IDs 960,989 and 1027804). Overall, an apparent downregulation of protein degradation mechanisms was observed in higher aluminum conditions. Moreover, silicon transporters (Protein IDs 912858, 1020251, 1010885, and 8915) were weakly downregulated. dAnk-like proteins (Protein IDs 1044363 and 1003123), which are believed to regulate phase separation between the organic matter and silica, were upregulated. Inorganic phosphate transporters (Protein IDs 1036788, 883475, 915403, 915394, 1389917, 902258, 846407, 1015189, 784627, and 1070080) were downregulated.

**Fig. 4:**
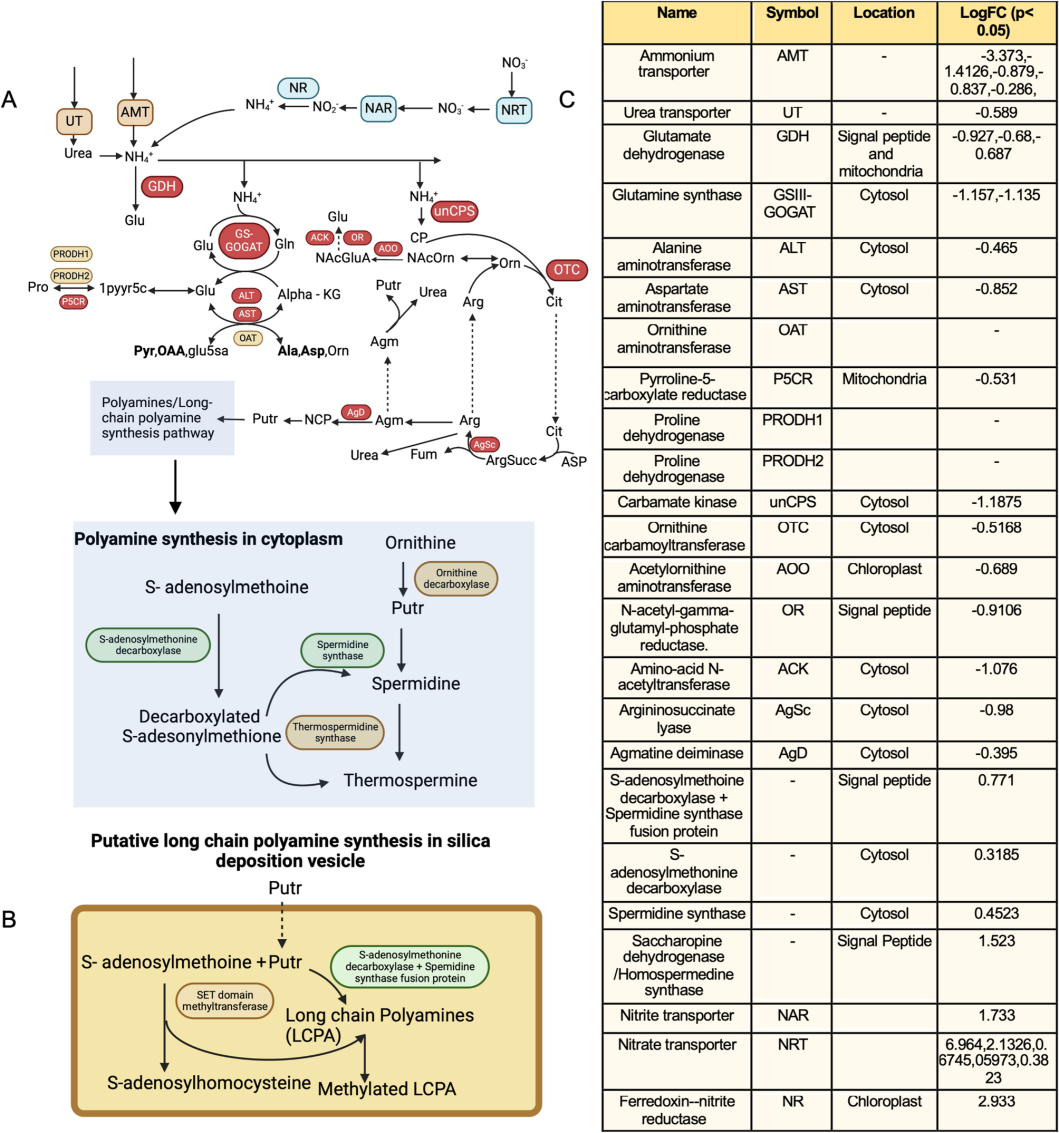
**A** Putative mapped nitrogen metabolism pathway in the diatom Entomonesis vertebralis and transcriptional responses of this pathway when aluminum was added. Red indicates the downregulation of an enzyme, and green represents the upregulation of an enzyme in response to aluminum. An overall increase in nitrogen uptake can be seen by mapping the transcriptional response of nitrate and nitrite transporters along with upregulation of nitrite reductase, indicating more ammonium is being produced in presence of aluminum. However, downregulation of 3 ammonium pathways, i.e. conversion into glutamate by GDH, the GS-GOGAT cycle, and the urea cycle, in addition to the upregulation of polyamine synthesis and long chain polyamine synthesis, can be noted. Glu – glutamate, Gln – glutamine, Ala – Alanine, Asp – Aspartate, Pyr – Pyruvate, OAA – Oxaloacetate, glu5a – gflutamate-5-semialdehyde, NCP-N – Carbamoyl-putrescine, NAcOrn – Acetylornithine, Orn – Ornithine, NAcGluA – N-acetylglutamate semialdehyde, Arg – Arginine, Cit – Citrulline, CP – Carbamoyl phosphate, Putr – Putrescine, Agm – Agmatine, 1pyr5c – pyrroline-5-carboxylate, Pro – proline, Alpha-KG – Alpha-Ketoglutaric acid, Fum – Fumarate. **B** Putative long chain polyamine synthesis pathway, illustrating the fusion protein that was upregulated in the presence of aluminum. C) Log-fold changes of expression of genes involved in this pathway in 10 μM aluminum compared to 0 μM aluminum

As certain metabolic processes were affected by the presence of aluminum, we investigated its effect on core metabolism, such as the nitrogen/ammonium assimilation and carbon metabolism pathways. The nitrogen metabolism pathway, as shown in Fig. 4, was constructed using the framework outlined by Smith et al. 2019 [34]. While not all genes within this pathway exhibited differential regulation, some genes involved in ammonium assimilation were downregulated.

Ammonium assimilation, as shown in Fig. 4a, primarily occurs through three distinct pathways: direct reduction of ammonium to glutamate by a glutamate dehydrogenase; the GS-GOGAT cycle, which involves glutamate synthase and results in the production of alanine, asparagine, and ornithine; and the urea cycle, which generates fumarate, putrescine, and urea; as outlined by Smith et al. [34]. These pathways can be predicted to be localized to mitochondria, chloroplasts, and other cell compartments (genes without mitochondria/chloroplast targeting sequences), as shown in Fig. 4c. In the current transcriptomics analysis, most of these transcripts involved in nitrogen metabolism lacked clear mitochondrial or chloroplast targeting sequences, suggesting that these proteins are not targeted to either organelle, as shown in Fig. 4. Notably, the differentially expressed glutamate synthase appeared to lack mitochondria or chloroplast targeting sequences and was determined to be unique to *E. vertebralis,* as it does not share similarity to other known diatom glutamate synthases from MCL clustering. Moreover, despite the upregulation of nitrate transporters and a nitrate reductase in the presence of aluminum, a paradoxical downregulation was observed in the ammonium assimilation pathway, as shown in Fig. 4a. Differential expression of genes possibly involved in polyamine synthesis was also observed, as shown in Fig. 4b. Furthermore, a significant downregulation was noted in the transmembrane amino acid transporter family 2.A.18.6.2.

**Fig. 5:**
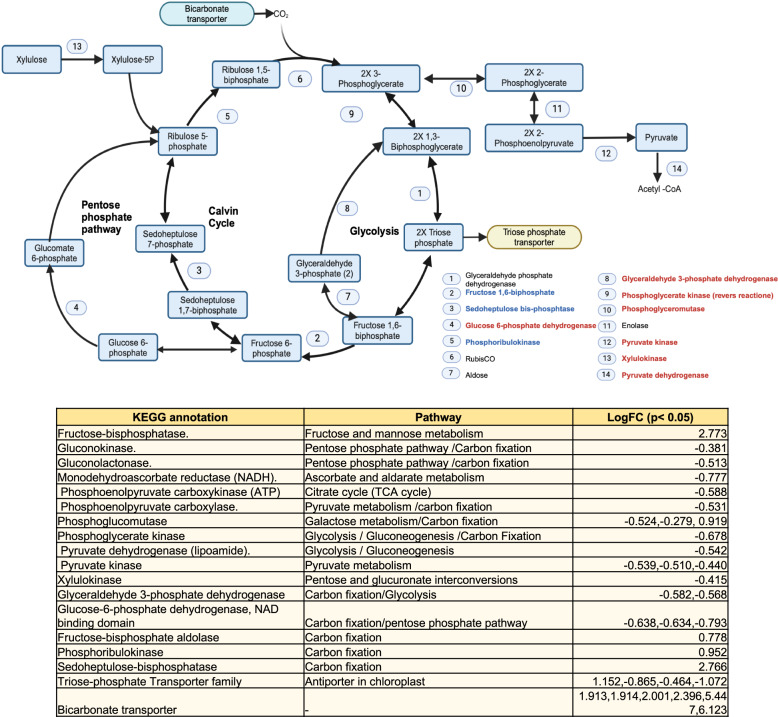
**A** Response of carbon metabolism of *E. vertebralis*, particularly the Calvin cycle, to the presence of aluminum. An overall upregulation of genes involved in the Calvin cycle was observed. Genes involved in the cycle such as fructose 1,6-biphosphate, sedoheptulose bis-phosphate, and phosphoribulokinase were upregulated. In addition, increased transcription of bicarbonate transporters was observed, indicating increased carbon fixation. However, downregulation was observed of pathways involved in glycolysis and the pentose phosphate pathway, which are ATP and NADH generating pathways, respectively. **B** LogFC of genes involved in carbon metabolism in 10 μM aluminum compared to 0 μM aluminum

Figure 5 provides an overview of the differential regulation of diatom genes related to carbon metabolism in the presence of aluminum. Notably, there was a pronounced upregulation of genes associated with the Calvin cycle, aligning with the concurrent upregulation of bicarbonate transporters when aluminum was present. This suggests an overall augmentation of carbon fixation (increased carbon uptake per cell) processes during aluminum supplementation. However, a subtle downregulation was observed in genes linked to the ATP-generating phase of glycolysis, which contrasts with the heightened energy-intensive carbon fixation.

Increases in diatom silicification and carbon uptake would likely require relatively more energy. Therefore, we investigated energy metabolism by characterizing whether photosynthetic genes such as some cytochrome b (Protein IDs 1058478 and 999804) and c (Protein ID 1058093) were upregulated in the presence of aluminum, which could indicate an increased electron transfer activity associated with photosynthesis. In addition, the upregulation of chlorophyll-binding proteins (Protein IDs 1050897 and 1059248) was observed, indicating an overall increase in photosynthetic activity. High upregulation of the ferritin-like diiron domain-containing protein (Protein ID 1076983) was also observed, which could be predominately used to store ferric ions mediating photosynthesis. Lastly, genes involved in lipid degradation metabolism were strongly differentially regulated in the presence of aluminum. Genes involved in glycerolipid synthesis such as glycerone kinase (Protein ID 986559), monogalactosyldiacylglycerol synthase (Protein ID 1006071) and glycerol-3-phosphate acyltransferase (Protein ID 1009955) were upregulated, although the triacylglycerol lipase involved in this pathway was downregulated (Protein ID 1083519). Increased upregulation of glycerone kinase and differential regulation of specific triglycerol lipases (Protein IDs 1034266 and 1083519) were downregulated, and triglycerol lipases of Protein IDs 1021083 and 797622 were upregulated [35]. Hence, differential regulation of different enzymes in lipid metabolism has been observed.

### Protein motif analysis identifies genes that are characterized by motifs involved in silicification

**Fig. 6:**
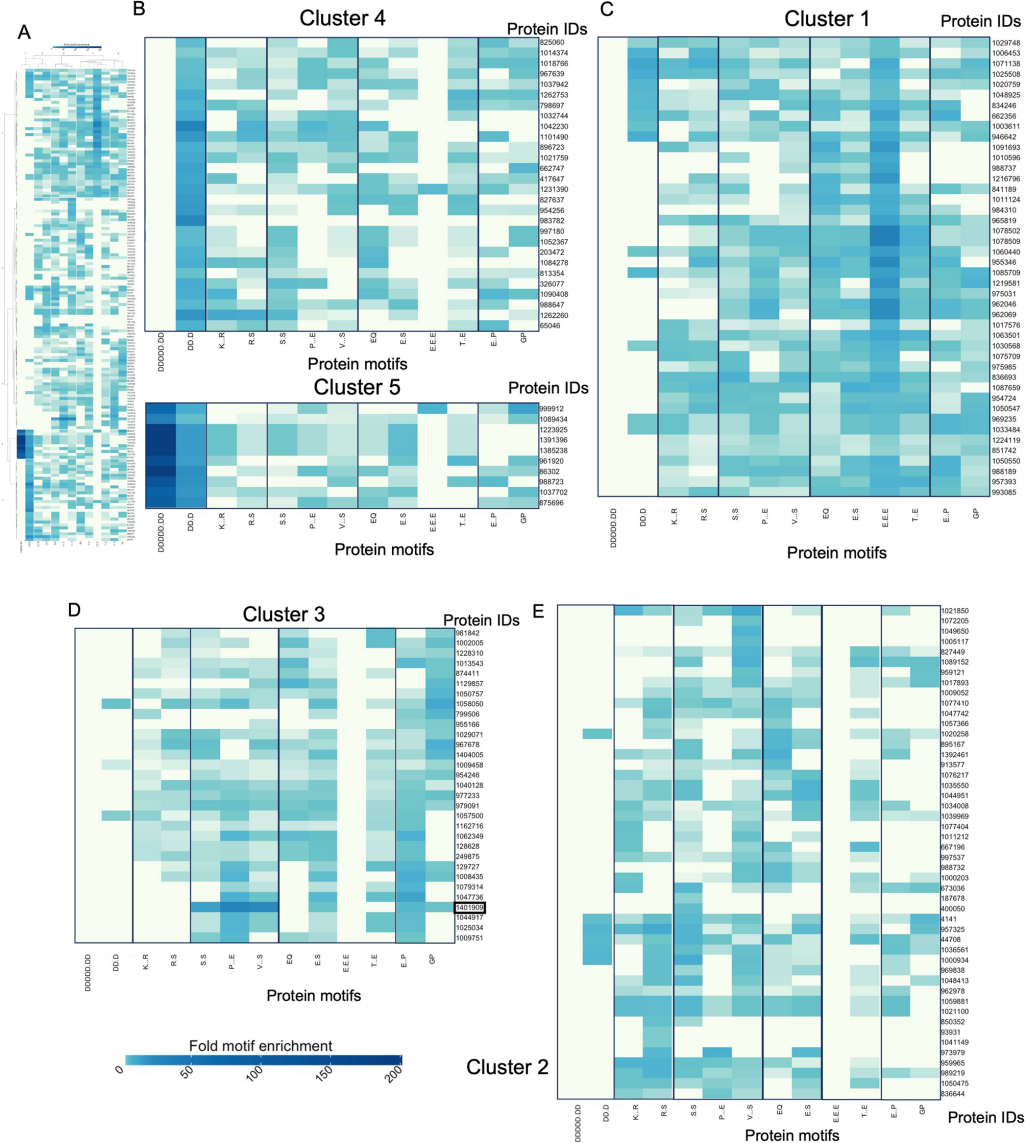
Transcriptomics on *E. vertebralis* when exposed to aluminum revealed proteins with unknown annotation that are characterized by silicification-based amino acid motifs. **A** Heatmap showing genes that were differentially regulated in *E. vertebralis* when exposed to aluminum without known annotation, and clustering based on enrichment of protein motifs. The heatmap was divided into 5 major clusters, numbered based on their position in the heatmap. A protein motif finder (ProminTools) was used for this analysis. The various clusters indicate enrichment in specific motifs relative to the background, i.e. the entire predicted proteome. **B** Clusters 4 and 5 showing enrichment in aspartic acid based motifs. **C** Cluster 1 showing enrichment in glutamic acid based motifs. **D** Cluster 3 showing an unusually enriched protein in serine-based motifs that suggests its involvement in silicification. **E** Cluster 2 showing all other proteins, some of which are enriched for lysine-based motifs. The right margin indicates the protein ID based on JGI annotation. Several unannotated proteins show enrichment in silicification-associated amino acids, such as serine, aspartic acid, and glutamic acid. In particular, Protein ID 1401909, which belongs to cluster 3, is highly enriched in serine motifs

While almost 50% of the differentially regulated diatom genes have annotations based on sequence, a notable portion lacks confident annotations or remains unannotated. The unannotated genes for which expression was associated with the increase in silicification between 0 μM and 10 μM aluminum were analyzed for unusual enrichment of particular amino acid patterns (referred to as motifs) relative to the entire proteome using ProminTools by Skeffington et al. [36], as shown in Fig. 6. Proteins were clustered based on the degree to which a specific motif repeats [36]. In addition, clustering was restricted to proteins that display a high degree of amino acid compositional bias (fLPS p-value > 10^–6^). Figure 6 shows enrichment of various motifs among selected genes that were differentially regulated in the presence of aluminum. The color intensity in the heatmap denotes enrichment of certain motif folds. Motifs involving serine and glutamic acid (Cluster 1), can be observed in Fig. 6c. Unusually high enrichment of repeating serines can be seen in Cluster 3 (Fig. 6d). This distinctive enrichment pattern led us to identify protein 1401909, which, through motif analysis, reveals a repeating motif of SEV.S.SE (Ser-Glu-Val-X-Ser-X-Ser-Glu-X). Notably, one of the most intensely enriched motifs, which is depicted by darker shades of blue, originates from Cluster 4 (Fig. 6b) and is primarily characterized by aspartic acid-based motifs. Enrichments of serine and the negatively charged amino acids such as aspartic acid and glutamic acid are observed among the unannotated differentially expressed genes.

### Structure-based annotation identifies sequence-divergent proteins with a conserved structure core

**Fig. 7:**
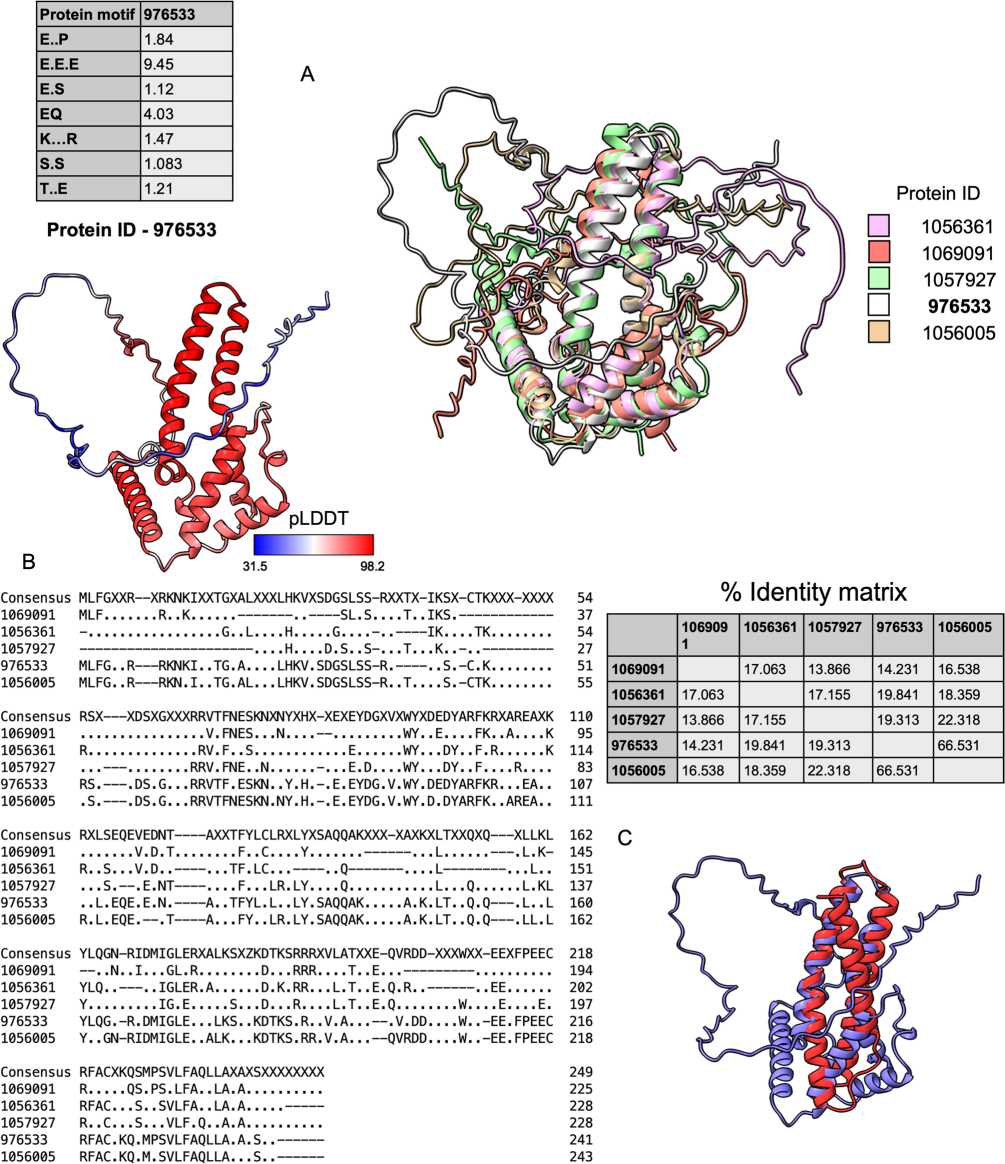
Structure-based protein families were revealed using transcriptomics of *E. vertebralis* when exposed to aluminum. **A** Structural superimposition of proteins belonging to a group of proteins that share structural similarity but are divergent in sequence. All of these proteins possess a structured tail and a disordered head. However, they do not share significant sequence-based similarity, even among the structured tail. **B** Depicting sequence-based alignment of all proteins belonging in the family. They share minimal sequence identity except for two members of the cluster. **C** The first hit from a database called CATHDB, depicts the structural similarity of the structured tail with cytoplasm elements, such as actin (red). All structures were predicted using Alphafold (see Methods). Using structural similarity, novel proteins families in response to aluminum were discovered

Several protein families associated with diatom silicification are poorly conserved across species [28]. Furthermore, conventional functional annotation relies on sequence-based similarity employing methods like BLAST and HMMER. As a complementary approach, we leveraged structure-based predictions for diatom proteins that were differentially expressed but lack any sequence-based annotation based on the JGI annotation pipeline [32]. We performed structure-based homology analyses to resolve potential functions of the unannotated genes identified in this study. Structure-based clustering suggested some unannotated proteins were sequence divergent, but shared predicted structural features. As shown in Fig. 7, one of the structure-based clusters that showed the highest upregulation (Protein IDs 1069091, 1056361, 1057927, 978533 and 1056005) was predicted to fold into a conserved structured domain consisting of 5 alpha helices and a disordered head. The low confidence in predicting the unstructured chain might have contributed least when seeking structural similarity.

This structure-based protein class lacks sequence similarity, as shown in Fig. 7b. Employing foldseek against the CATH database revealed a predicted relatively low similarity (0.15 e-value) to certain cytoplasmic elements, such as actin and microtubules, as depicted in Fig. 7c. These sequences eventually fold into certain conserved sequences and could share similar function independent of the sequence.

## Discussion

In this study, cultures of the diatom *E. vertebralis* were exposed to different concentrations of aluminum and their cellular responses were monitored by using differential transcriptomics. Previous studies have observed that aluminum enhances metabolic pathways such as carbon fixation [14, 37], which could be due to improved bioavailability of iron in the form of Fe^2+^. Our current study supports the hypothesis that aluminum increases iron bioavailability, as we observed an upregulation of gene encoding ferritin-domain-containing proteins involved in iron storage in pennate diatoms [38]. Aluminum exposure resulted in an apparent downregulation of inorganic phosphate transporters consistent with [21], which suggests enhanced phosphate metabolism, particularly that of dissolved organic phosphate.

Additionally, we mapped responses within pathways involved in nitrogen/amino acid metabolism based on genes that were differentially regulated in the presence of aluminum. While there appeared to be an upregulation of nitrate transporters and nitrate reductase, which convert nitrates to ammonium, there was an overall transcriptional level decrease in genes involved in nitrogen assimilation. Most of the differentially regulated genes in nitrogen assimilation appeared to be lacking in chloroplasts and mitochondria targeting sequences, indicating that differentially expressed nitrogen assimilation may occur in the cytoplasm; however, additional experiments are required to verify this. The upregulation of the nitrate transporters and nitrate reductase might imply nitrogen limitation, which has been previously observed; however, we did not observe a downregulation of the Calvin cycle, thus differing from the nitrogen limitation response in Bender et al. 2014 [39]. In addition, we identified a glutamate synthase unique to *E. vertebralis,* which appears to lack clear mitochondrial and chloroplast targeting sequences and is possibly localized in the cytoplasm. Furthermore, a family of transmembrane amino acid transporters (TCDB ID 2.A.18.6.2) were downregulated; these proteins could be responsible for transporting glutamate from the cytoplasm to the mitochondria. In general, a decrease in ammonium assimilation was observed; however, the genes that were differential regulated appears to lack clear mitochondrial and chloroplast targeting sequences and is possibly localized to the cytoplasm. This nitrogen assimilation pathway may have been derived from recycling protein-derived nitrogen, which is concurrent to claims proposed by Bernard et al. 2009 [40].

The observed upregulation of genes in the Calvin cycle in the presence of aluminum aligns with previous findings, indicating enhanced carbon uptake [37]. Notably, the Calvin cycle is known to require extensive energy. A plausible response to the increase in required energy is an increase in photosynthesis, possibly attributed to heightened iron bioavailability. The concurrent upregulation of cytochromes, facilitating electron transfer mechanisms, supports this hypothesis. However, a crucial question arises regarding the fate of carbon. Contrary to expectations, the downregulation of the latter half of glycolysis, responsible for ATP synthesis, challenges the notion of glucose being the primary energy source. Intriguingly, the pronounced upregulation of lipases and glycerone kinase points towards lipid metabolism as an alternative route for carbon assimilation and energy acquisition. Upregulation of glycerone kinase suggests the upregulation of glycerol metabolism, which could be produced from certain types of lipids [41, 42]. Tanaka et al. 2015, reported that increased lipid accumulation in *Fistulifera solaris* was linked to an upregulation of the fatty acid degradation pathway and a concomitant downregulation of ammonium assimilation [43]. This leads us to hypothesize an elevated production of lipids in response to aluminum-induced stress. Our observations suggest an association between aluminum stress and lipid metabolism alterations. The upregulation of rate limiting glycerol-3-phosphate acyltransferase [44] and monogalactosyldiacylglycerol synthase, both of which are involved in glycerolipid biosynthesis, strengthens the claim that the presence of aluminum could alter lipid production.

One potential confounding variable in the above carbon and nitrogen metabolism analysis is the slight difference in cell density observed between the different culture conditions, with the addition of aluminum leading to a reduced cell count (Fig S4). However, Bsi analysis shows that the total amount of silica consumed from the media in each condition is not significantly different (see following paragraph). This reduction of cell count is proportional to the increase in silica uptake per cell (Fig. 2a) indicates that silicon is the limiting nutrient. Additionally, as f/2 media generally contains an excess of macronutrients (nitrogen and phosphate) relative to silica, and since the rate of silica depletion does not significantly differ across aluminum concentrations, the depletion of other nutrients in the culture was assumed to be similar across conditions, with concentrations still in excess at the time of sampling. However, differences in biomass density could cause changes to other media attributes such as pH, which could have an impact on nutrient uptake.

**Fig. 8:**
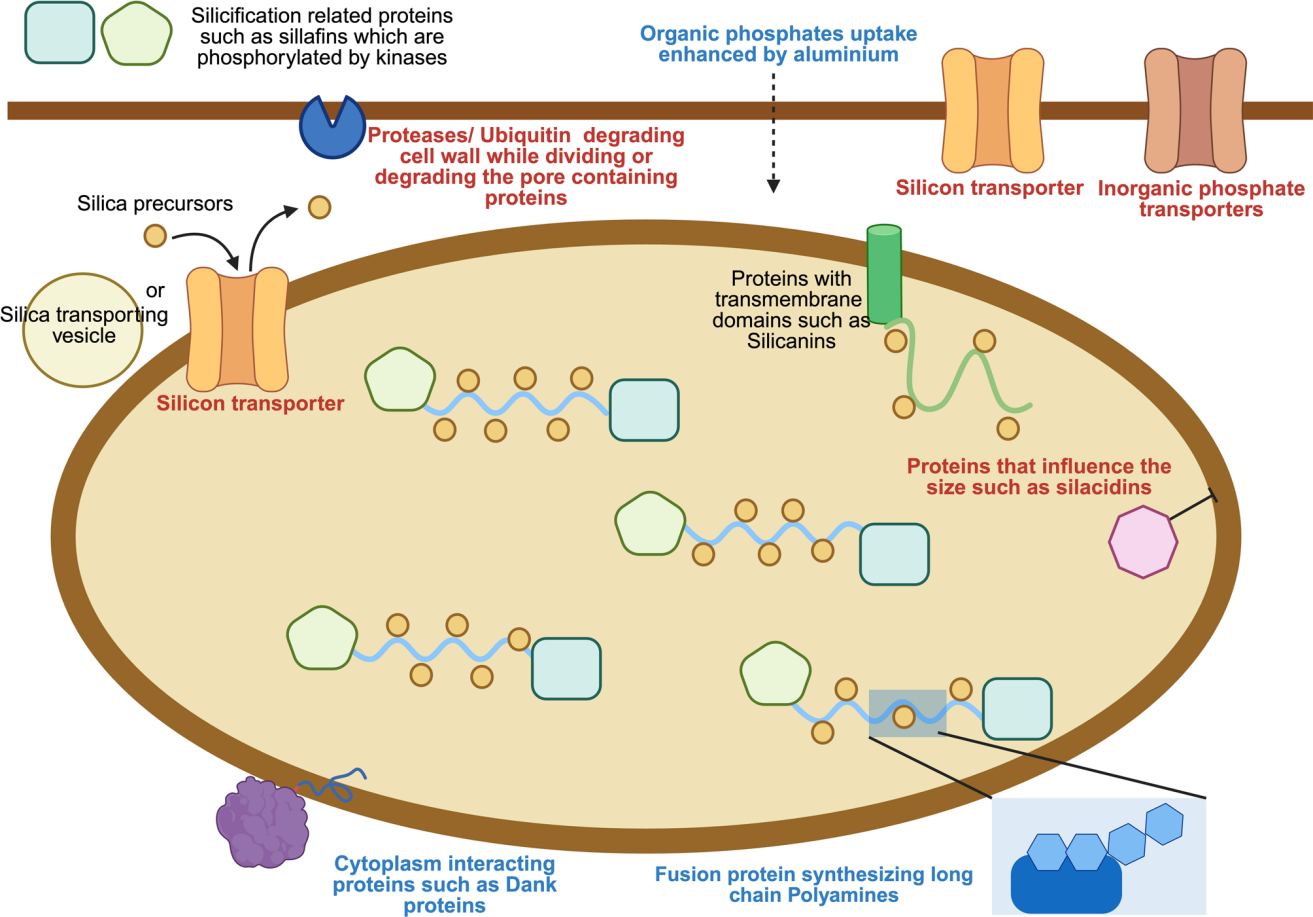
Putative elements involved in diatom frustule formation or in aiding frustule formation. The model involves different pathways, such as proteolysis to break down proteins involved in pores in the cell wall; fusion proteins for synthesizing long-chain polyamine, which are engaged in silica precipitation; proteins that interact with cytoplasm, such as the dank proteins; and the proteins that are assumed to be involved in silicification, such as silicadins, or involved in transporting the silica precursors, such as the silicon transporters. Various elements are differentially expressed in the presence of aluminum, which may result in frustule morphology change. Red indicates downregulation and blue indicates upregulation in presence of aluminum

We observed that exposing *E. vertebralis* to higher aluminum concentration increases the biogenic silica content per cell and changes the frustule morphology, as observed by TEM. In addition, transcriptomic analyses suggest that the genes involved in silicification were subjected to differential regulation, which has been summarized in Fig. 8. Silicon transporters (SITs) are believed to be involved in silicic acid transport when it is deficient in the cellular environment [45].

When excess silicic acid is present in the cellular environment, some of these transporters have minimal to no roles in transport, but may instead sense extracellular silicic acid levels and allow the cell to evaluate whether it can proceed for cell division [46]. However, an estimate of the available silicic acid at the time when the cultures were harvested, based on the mass balance between the initial silicic acid concentration and the amount of biogenic silica present at the time of sampling, indicates that around 56 μM of silicic acid was available for diatoms exposed to aluminum compared to ~ 64 μM for those in controls (no aluminum). Both of these concentrations are greater than 30 μM, which has been previously proposed to be a threshold above which certain silica transporters are upregulated and, suggesting that cells in both treatments may rely on diffusion rather than SIT-meditated silicic acid transport. The amount of silica available per cell in aluminum-exposed diatoms was higher than that of the control, so downregulation of SIT’s transcripts might be as a mechanism for sensing and evaluating the needs of silicic acid. In addition, the downregulation of silicon transporters in the presence of aluminum, despite higher levels of available silica per cell, is consistent with evidence from higher plants where genes encoding silicon transporters are downregulated in plant shoots with higher silica accumulation levels [47]. We hypothesize that to detoxify aluminum, the cell incorporates the metal into their frustules [48], and the silicon transporters could be signaling the cell for increased silica requirement by downregulating its expression. However, more studies are needed to explain this molecular mechanism of silicon transporters.

Silacidins and silaffins, which have been implicated in diatom silicification, can undergo proteolytic cleavage [24, 49]. As previously reported by Shrestha et al. [50], protein degradation is crucial for pore formation and rearrangement of cell wall components during valve synthesis [50]. Downregulation of most of the ubiquitin-ligases [50] and certain proteases could be an indication of decreased protein degradation in the presence of aluminum. Proteins and long-chain polyamines together form the organic phase that could control the silica polymerization via liquid–liquid phase separation, as proposed by Sumper 2002 [51]. The size of the peptides that form this organic phase could play an essential role in controlling the size of the cell and pores. In addition to these peptides, dAnk proteins [52] were recently identified as key players in pore formation by regulating the phase separation of the organic phase. The upregulation of dAnk proteins could indicate that the cell controls this phase separation process to mediate silicification. Although strong upregulation of dAnk proteins and protein degradation mechanisms was seen, this strain of *Entomoneis vertrbralis* did not exhibit much pore-type morphology. Moreover, upregulation of a fusion protein, which has been hypothesized to be involved in the synthesis of long-chain polyamines, was upregulated in the presence of aluminum, suggesting that increases in long-chain polyamine production could also contribute to increases in silicification [53]. In conclusion, although differential regulation of conventional genes involved in silicification was not observed in this study, differential regulation was observed for other mechanisms related to silicification, such as protein degradation, synthesis of long-chain polyamines, and organic phase control. Further research is required to improve the mechanistic understanding of how these processes affect the degree of diatom silicification.

Conducting RNA-seq on aluminum-exposed diatoms provided opportunities to identify genes involved in morphological changes or differences in the diatom’s frustule. *E. vertebralis* is only significantly silicified in the region of the raphe in the form of circular rings (Fig. 1), making it a good candidate for linking the differential expression of genes to specific phenotypic changes or differences in raphe morphology. The identification of genes involved in silicification is challenging because it appears that, based on sequence alone, these genes are poorly conserved among and within species and are poorly characterized [28]. We hypothesize that genes that lack sequence conservation may still share conserved structural elements. Using structure prediction, a structure-based family of proteins was identified that shares a structured tail but does not share much sequence similarity. Some of the silicification-associated proteins, such as the dAnk-like proteins, have some cytoplasm-interacting domains that are structured and simultaneously have similar unstructured tails that could mediate silicification. A similar pattern is observed in this structure-based protein class, which shares some structural similarities with known cytoplasmic protein domains such as microtubules, albeit with low confidence. As one of the proteins in this cluster shares weak sequence similarity to an uncharacterized protein that is present in other sequenced pennate diatoms and is upregulated in cells that have increased silicification, it may be a pennate-specific silicification-associated protein. We speculate that this protein cluster could be involved in establishing a connection between the silica deposition vesicle and cytoplasmic elements that could guide the cell to synthesizing an asymmetric pennate-specific frustule.

**Fig. 9:**
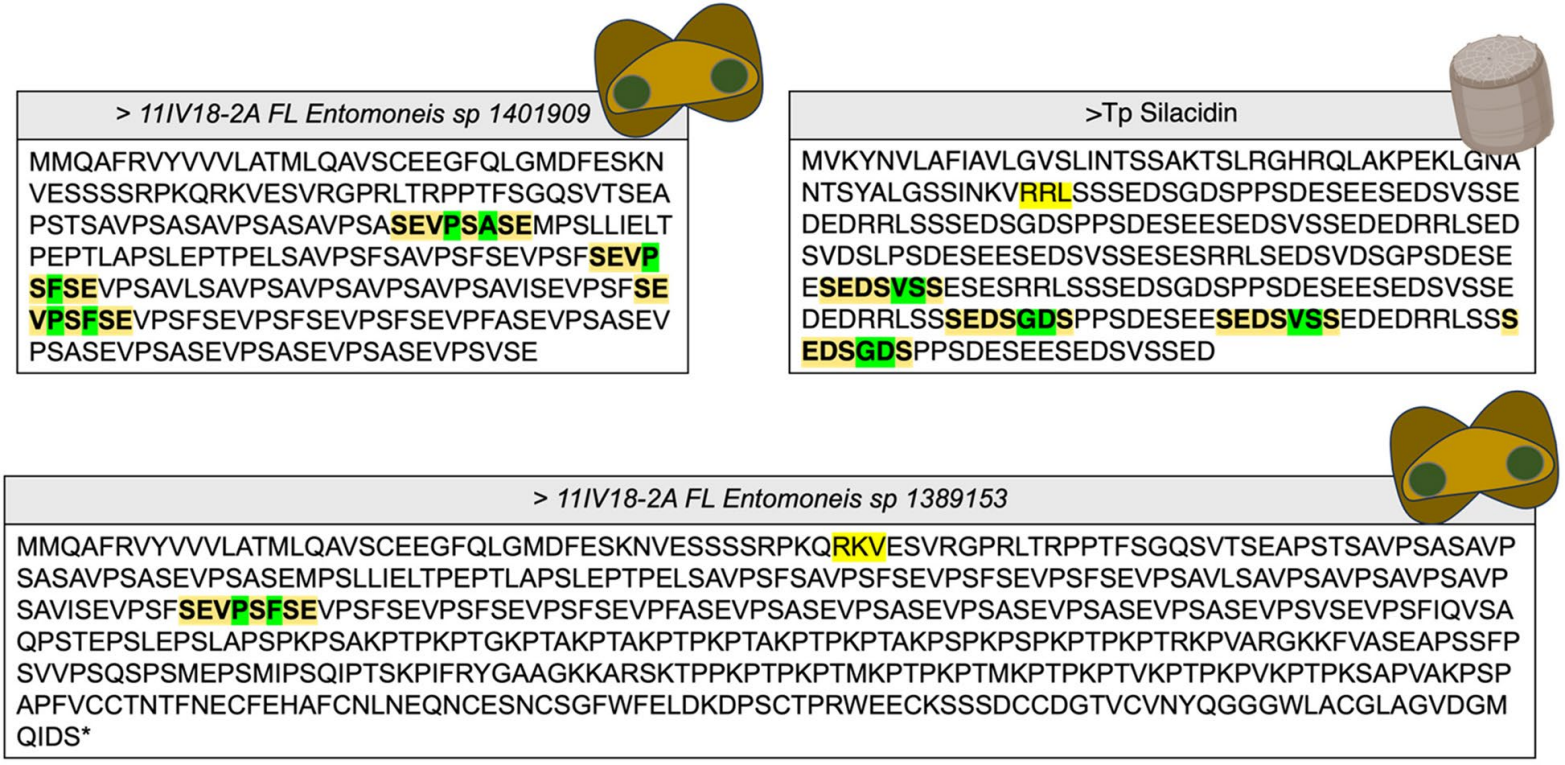
Protein ID- 1401909, which has unusual enrichment of serine-based motifs from Fig. 6. By further analysis, this protein has different gene models, additional experiments are required to verify which gene moedel is correct.Different gene models of the protein of interest putatively involved in silicification (1401909 Vs 1389153) and their comparison with Tp Silacidin is shown in this figure. Both gene models have SEV.S.SE as a repeating motif, which is similar to a known silicification-associated protein (Silacidin) in T. psedonana, which is known to control the size of the cell. Alternative gene model unusually repeating lysine and proline, which is known characteristics of silicification associated proteins

Many of the unannotated genes identified in this study appear to be enriched in serine-containing amino acid motifs. Patterns of serine have been associated with silicification [54], but little is known regarding the function of these repeats. However, it is known that serine and tyrosine can undergo post-translational modifications where the hydroxyl group is modified with a negatively charged phosphate. It has been reported that silicification-associated proteins that lack phosphate modifications do not catalyze silica precipitation [54]. We speculate that serine-containing protein motifs might have a putative role in catalyzing silicification. As shown in Fig. 9, we identified protein ID 1401909, which appears unusually enriched in serine moieties. Upon further analyses of this gene product, it has a highly repetitive SEV.S.SE motif. The putative gene model contains additional repetitive PT and K…K motifs, which are known to be associated with silicification [55]. This protein sequence shares similar repeating patterns of serine as seen in silacidin from *T. pseudonana* [56], which is also enriched in a similar motif SEDS. SE; however, the protein from *E. vertebralis* does not share any sequence similarity to the *T. pseudonana* silacidin. Previously, downregulated silacidin was reported to correlate with increased cell size in *T. pseudonana* [56, 57], which is consistent with what we observe, as this particular gene is highly downregulated in the presence of 10 μM aluminum, which causes larger rings to protrude out of the raphe in *Entomoneis vertebralis.* Exposing the *E. vertebralis* to higher aluminum concentrations—caused increased size of the rings associated with the raphe, which could account for the increased silicification caused by aluminum.

The artificial seawater is not completely lacking aluminum, the control cultures also contain some amounts of aluminum. Quantitative analyses suggest that the molar ratio of aluminum/silicon in *E.vertebralis* remained approximately constant across varying aluminum concentrations, mirroring trends observed previously in a different diatom strain [37]. This suggests that the observed increase in silicification and incorporation of aluminum could be a way to alleviate aluminum stress and toxicity, as has been observed in plants that embed aluminum in silica nanospheres [22]. Additionally, the overall increase in dissolved organic phosphates, carbon, nitrogen, and iron caused an increase in general metabolism, which may account for enhanced bio-silicification.

## Conclusion

Overall, our results characterize and correlate the diatom’s transcriptional responses and frustule morphology changes when exposed to aluminum. The transcriptional response to aluminum provides new insights into the different metabolic responses of diatoms to aluminum. In addition to altered frustule properties, the impact on core metabolism has been shown to have some beneficial effects such as increased carbon fixation, which will help us engineer diatoms to produce valuable products and understand their ecological importance. In combination with state-of-the-art computational tools, such as structure-based similarity and protein motif analyses of differentially expressed genes, we have identified a set of proteins that could be directly or indirectly impact diatom silicification. These results advance our understanding of the factors influencing diatom metabolism, silicification, and production of other biosynthetic compounds, paving the way for the identification of new metabolic targets for engineering or controlling biosynthetic processes.

## Materials and methods

### Cultivation of diatoms

*Entomoneis vertebralis* (UTEX LB 3214) was received from the UTEX Culture Collection of Algae and cultivated in half salinity artificial seawater medium (~ 15 psu) supplemented with f/2 additives (ASW-f/2) in 14:12 day light cycle [29]. Prior inoculation, media was pH balanced to 8.2. Precautions were taken to minimize the alterations of the f/2 trace metals and aluminum concentrations during preparation of the media. Specifically, all additives—nitrate, phosphate, silicate, trace metals, vitamins, and aluminum—were filter-sterilized rather than being autoclaved. This strain may also go by *Entomoneis* sp. (11IV18-2A), based on their isolate number. Aluminum chloride hexahydrate (99.8%) was added to the media to achieve added ­Aluminum concentrations of 0.2 µM, 1 µM, 2 µM, 5 µM, and 10 µM. Three biological replicates were grown. A control culture was grown without ­Aluminum addition. Culture flasks were shaken manually every single day to assure proper mixing of the medium. Cell counts were taken manually using hemacytometer (HS-3510). After 22 days, cells (9 ml culture) were harvested through centrifugation at 3000 g for 10 min to isolate RNA. After centrifugation, an equal amount of RNA later was added and removed using centrifugation (3000 g for 10 min). The samples were flash-frozen in liquid nitrogen and stored at −80 °C. For Biogenic silica analysis, cells (0.25 ml) were collected on a 0.6 µm polycarbonate filter through vacuum filtration and frozen at −20 °C for further analysis.

### RNA isolation and data analysis

Total RNA was isolated using a Zymo Quick-DNA Fungal/Bacterial Miniprep Kit. The cells stored in RNA later were lysed in a bead beater (30 s bead beating, 2 min ice for 4 cycles) followed by the instructions in the kit. RNA was eluted in 50 ul of RNase-free water and then re-eluted through the column to concentrate the RNA and stored at −80 ^0^C until further analyses. RNA concentration was checked on the Invitrogen Qubit 2.0 fluorometer. Quality was evaluated with an Agilent 2200 TapeStation or 2100 Bioanalyzer – all samples had a RIN or RINe score above 7. The NEBNext Ultra II Directional kit was employed to prepare RNA for sequencing, following the manufacturer’s instructions. RNA was sequenced on a NextSeq 500 platform using a 75 Cycle High Output run on single end mode. Raw reads were analyzed for quality using FastQC v 0.12.1 [58], based on that, trimming and further cleaning was designed. Raw reads were trimmed, and adapter sequences were removed using fastp (setting -q 15) [59]. The reference genome was accessed through the JGI portal, PhycoCosm [32] under the name of *Entomoneis* sp. UTEX 11IV18-2A FL. Reads were aligned to the reference using HISAT2 (-p 12 –dta mode) [60]. Output files from HISAT2 (SAM files) were sorted and indexed using SAMtools v. 1.7 to BAM files [61]. Further, transcripts were assembled using StringTie v2.2.1 (settings –p 20) [31]. The assembled transcripts generated by StringTie were merged in order to account for de-novo transcripts and used as a reference genome to calculate the gene count matrix. Genes with CPM > 0.5 in at least one condition were considered for further analysis. Further, the gene count matrix was analyzed using edgeR [62] for differential gene expression analysis. Voom [63] was used to calculate the mean–variance trend, which was further used by limma [64] to create contrasts between different conditions. For further analysis, genes that are differentially regulated with a p-value less than 0.05, were considered.

Sequence-based annotations were imported through JGI PhycoCosm [32], which included SignalP [65], TMHMM [66], InterPro [67], Uniprot [68], KEGG [69] and KOG [70]. KEGG and KOG annotations were used to explore nitrogen and carbon pathways. Chloroplast/mitochondria localization prediction was done using HECTAR [71]. For structure-based analysis, the protein structures of the genes that did not have any annotation (from above) were predicted using AlphaFold 2.3.1 [72]. Further the Alphafold [72] code was modified to run on using parallel computing. Alphafold provides, 5 possible models for a given protein sequence, Model 0 which has the highest confidence among all the predicted models, for each protein was chosen for further analysis. To predict functionality of predicted protein structures and cluster them based on strucrure, Foldseek was employed [73]. Foldseek was installed on local computing cluster using the AlphaFold proteome and the Protein Data Bank (PDB) as a reference database. In addition to it, a database of alphafold predicted known silicification genes. For structural similarity, Foldseek with the easy-search option was used, and for creating structure-based clusters, the easy-cluster option was used with c −0.9 setting. The per-residue local distance difference test (pLDDT) confidence scores for the protein structure models were retrieved from the B-factor field of the coordinate section of the output pdb file, and the final structures were visualized and aligned using UCSF ChimeraX. Protein Motif analysis was conducted using ProminTools [36], with fLPS p-value > 10^–6^, using the predicted protein in the genome as the background proteins. Protein clustering performed by JGI computed based on the TRIBE-MCL clustering method [74] was used to identify genes specific to diatoms by comparing them with related Ochrophyta.

### Biogenic silica analysis

Biogenic silica analysis was conducted according to Krause et al. 2009 [30]. Cells were collected on 0.6 µm polycarbonate filters, dried for 2 days, and dissolved in 0.2 M NaOH. Blank tubes with filter were also included in these treatments to account for background interferences. This dissolution of silica was carried out at 95 °C for 1 h and then immediately placed in ice. Excess of NaOH was neutralized by adding 1 N HCl. The dissolved sample was centrifuged to remove particulates. The supernatant was diluted, and 4 ml of 6.87 mM of ammonium molybdate (8 g ammonium molybdate in 1000 ml Nanopure water, when dissolved, 24 ml concentrated HCl were added). After 10 min, excess of ammonium molybdate that did not bind to silica was reduced by adding a 6 ml reducing agent. The reducing agent was made by combining 4 solutions: 2 ml Metol-sulfite reagent with 1.6 ml Nanopure water, 1.2 ml 50% H2SO4, and 1.2 ml saturated oxalic acid for each sample. Metol-sulfite solution was made by dissolving 12 g of sodium sulfite (Na2SO3) in 1000 ml Nanopure water. Once dissolved 20 g para-methylaminophenol sulfate were added. The samples were kept overnight and measured for fluorescence next day usinf UV spectrophotometer. Similarly, standards from 0.625 to 56.25 µM silica concentrations were prepared to make a calibration curve. Once the silica concentration was known, it was used to calculate silica per cell using the cell count taken just before the filtration.

### SEM and STEM on cleaned diatom frustules

50 ml of exponentially growing cultures were centrifuged (3000 g for 10 min) and further dissolved in 20 ml of 30% sodium peroxide at 60 °C for 1 h (diatoms must have turned white in color). Cleaned frustules were further washed with MilliQ water for 5 times. Further, the cleaned frustules were suspended in 70% ethanol and dried over the SEM stub. To isolate the raphe from the frustule sonication was performed for 2 min under 20% power using a Branson Tip Sonicator. The material was then sputter-coated with gold and imaged using Thermo Scientific Apreo C LoVac FEG SEM at 5 kV accelerating voltage.

Transmission electron microscopy (TEM) images were acquired using Talos F200X (Thermo Scientific) with an accelerating voltage of 200 kV. 10 μl of the diluted H_2_O_2_ cleaned frustules extracted from diatom cultures of different exposed aluminum concentrations were loaded on the 300-mesh lacey carbon Cu grid (Ted Pella), and the excess of water was removed using filter paper followed by drying in vacuum overnight. Scanning transmission electron microscopy (STEM) was performed on the same equipment in the high-angle annular dark-field (HAADF) mode.

### ICP-MS on whole cell to measure aluminum uptake

Cells of *Entomoneis* (11IV18-2A), grown under 0, 5, and 10 µM added aluminum conditions, were washed with 10 mM EDTA (pH 4.5), were collected on a 0.6 µm Hcl cleaned polycarbonate filter and dried overnight at 60 °C. These filters were digested by adding 2 mL of a mixture of concentrated strong acids (final concentrations: 4 M HCl, 4 M HNO₃, and 4 M HF) [75]; and heated for 4 h at 110 °C in a PFA bomb. The acid digest was transferred into another bomb, ensuring complete transfer by rinsing the primary bomb with DI water. The acid digest was further dried for 2–3 h at 110–115 °C. Then, 1 ml of HNO₃/H₂O₂ mix (50% HNO₃, 15% H₂O₂, and 35% DI) was added to the dried acid digest, which was dried further for 2–3 h at 110–115° C. After cooling down, the digest was reconstituted in 2% HNO₃ and transferred into ICP vials. Samples were analyzed using quadrupole ICP-MS (PerkinElmer NexION 350D) with an indium internal standard and quantification by external standard curve.

## Supplementary Information

Supplementary Material 1.

## Data Availability

RNA-seq data generated during this study are available on NCBI SRA under BioProject ID:PRJNA1258573. Alphafold code and structures are available on request. All the data generated during analysis is reported in supplementary information.

## Abbreviations

ICP-MS: Inductively coupled plasma mass spectrometry
HAADF: High- angle annular dark-field imaging
STEM: Scanning transmission electron microscopy
Bsi: Biological silica
CATH: Class architecture topology and homologous superfamily
BLAST: Basic local alignment search tool
HMMER: Hidden markov model
GS-GOGAT: Glutamine synthetase and Glutamine:2-oxo-glutarate aminotransferase
Pfam: Protein family database
KEGG: Kyoto encyclopedia of genes and genomes
